# Neurotrophic effects of progranulin *in vivo* in reversing motor neuron defects caused by over or under expression of TDP-43 or FUS

**DOI:** 10.1371/journal.pone.0174784

**Published:** 2017-03-30

**Authors:** Babykumari P. Chitramuthu, Denis G. Kay, Andrew Bateman, Hugh P. J. Bennett

**Affiliations:** 1 Endocrine Research Laboratory, Royal Victoria Hospital, McGill University Health Centre Research Institute, Montreal, Québec, Canada; 2 Neurodyn Inc., Charlottetown, Prince Edward Island, Canada; University of Michigan, UNITED STATES

## Abstract

Progranulin (PGRN) is a glycoprotein with multiple roles in normal and disease states. Mutations within the *GRN* gene cause frontotemporal lobar degeneration (FTLD). The affected neurons display distinctive TAR DNA binding protein 43 (TDP-43) inclusions. How partial loss of PGRN causes TDP-43 neuropathology is poorly understood. TDP-43 inclusions are also found in affected neurons of patients with other neurodegenerative diseases including amyotrophic lateral sclerosis (ALS) and Alzheimer's disease. In ALS, TDP-43 inclusions are typically also immunoreactive for fused in sarcoma (FUS). Mutations within TDP-43 or FUS are themselves neuropathogenic in ALS and some cases of FTLD. We used the outgrowth of caudal primary motor neurons (MNs) in zebrafish embryos to investigate the interaction of PGRN with TDP-43 and FUS *in vivo*. As reported previously, depletion of zebrafish PGRN-A (zfPGRN-A) is associated with truncated primary MNs and impaired motor function. Here we found that depletion of zfPGRN-A results in primary MNs outgrowth stalling at the horizontal myoseptum, a line of demarcation separating the myotome into dorsal and ventral compartments that is where the final destination of primary motor is assigned. Successful axonal outgrowth beyond the horizontal myoseptum depends in part upon formation of acetylcholine receptor clusters and this was found to be disorganized upon depletion of zfPGRN-A. PGRN reversed the effects of zfPGRN-A knockdown, but a related gene, zfPGRN-1, was without effect. Both knockdown of TDP-43 or FUS, as well as expression of humanTDP-43 and FUS mutants results in MN abnormalities that are reversed by co-expression of hPGRN mRNA. Neither TDP-43 nor FUS reversed MN phenotypes caused by the depletion of PGRN. Thus TDP-43 and FUS lie upstream of PGRN in a gene complementation pathway. The ability of PGRN to override TDP-43 and FUS neurotoxicity due to partial loss of function or mutation in the corresponding genes may have therapeutic relevance.

## Introduction

Progranulin (PGRN) is a secreted glycoprotein that regulates many processes including cell proliferation, survival and motility [1]. In mammals, it is composed of seven repeats of the disulfide-rich granulin motif. PGRN has potential therapeutic activity in neurodegenerative diseases since it has been found that viral vector gene delivery of *GRN* suppresses the development of disease-like phenotypes in murine models of Parkinson’s disease (PD) [2] and Alzheimer’s disease [3]. Haploinsufficiency of *GRN*, the gene encoding PGRN, results in 50% depletion of PGRN protein levels, and causes frontotemporal dementia (FTD), an early onset form of dementia [4, 5]. To date 79 pathological mutations in *GRN* have been reported (www.molgen.ua.ac.be/FTDmutations/) the majority of which result in nonsense mediated decay of the GRN mRNA transcript [4–6]. FTD is a general term for a group of disorders that are characterized by frontotemporal lobar degeneration (FTLD). This is a progressive, irreversible atrophy of the frontal and temporal cerebral cortices, regions of the brain that, among many functions, control planning, judgement, speech and some types of movement [7, 8]. About 30–50% of FTD cases are familial, and are most often caused by mutations within one of three genes; microtubule-associated protein tau (*MAPT*), *GRN* and hexanucleotide repeat expansions in chromosome 9 open reading frame 72 (*C9orf72*) [9–12]. *GRN* mutations are responsible for 5–10% of all cases of FTLD and 13–25% of familial cases [13]. The histopathology of FTLD is typically characterized by the presence of cellular inclusions. These are deposits of hyperphosphorylated tau-protein (FTLD-tau), in the case of mutations of *MAPT* [14], ubiquitinated inclusions (FTLD-U) containing carboxyl-terminal fragments of TDP-43 (TAR-DNA binding protein-43) (FTLD-TDP) in the case of mutations of *GRN* or *C9orf72* [15, 16]. TDP-43 proteinopathy is also common in the motor neurons (MNs) of patients with amyotrophic lateral sclerosis (ALS) [17], and in the brains of patients with Alzheimer’s disease (AD) [18]. In some cases of FTLD-U, the neurons exhibit ubiquitin inclusions that are negative for TDP-43, and in such cases the inclusions are often positive for FUS/TLS (fused in sarcoma/translocated in sarcoma) [19]. FUS-containing inclusions are also common in sporadic and familial ALS [20]. Mutations of either *TARDBP*, the gene encoding TDP-43 or *FUS* can cause either ALS [12, 21, 22] or FTLD [23] confirming the neuropathological roles of these two proteins.

Both TDP-43 and FUS are RNA-binding proteins. Other neurodegenerative conditions including spinal muscular atrophy are also caused by mutations within genes for RNA-binding proteins [24]. Understanding the pathological interplay between PGRN and TDP-43 is difficult since both molecules have highly diverse functions. PGRN is a neuronal survival factor [25–30], but also regulates important aspects of neuroinflammation [31–41] and modulates lysosomal function [42–44]. TDP-43 plays multiple roles in RNA processing [45], including the suppression of cryptic exons [46]. It is a component of the RNA-stress granule [47] and in this context, is important in the neuronal response to injury [48]. If, as we [25, 27] and others [28] propose, one function of PGRN in the brain is as a cell survival factor, the pathological consequences of PGRN depletion may be observed best in its interaction with other injurious stimuli. How PGRN, TDP-43 and FUS interact has been investigated following the neurotoxic expression of expanded polyglutamine (polyQ) tracts in the *huntingtin* gene. Both TDP-43 and FUS are required for full expression of polyQ toxicity in mammalian striatal neurons [49]. PGRN alleviates the enhancement of polyQ toxicity conferred by TDP-43. It has, however, no influence on the ability of FUS to worsen polyQ toxicity. Similar interactions of PGRN and TDP43 were obtained in a *C*. *elegans* model of MN toxicity caused by *huntingtin* exon-1 polyQ toxicity. These results support a genetic interaction between TDP-43 and PGRN in evoked neuronal cell death. This conclusion is based upon the fact that PGRN blocks polyQ toxicity and TDP-43 blocks the cytoprotective action of PGRN. However, no corresponding interaction between PGRN and FUS was observed.

It is unclear, whether the depletion of PGRN is by itself a direct cause of TDP-43 toxicity, or whether TDP-43 related neurotoxicity is a secondary response to a more generalized cellular dysfunction caused by PGRN depletion. Zebrafish provide a vertebrate model system in which gene complementation studies, similar to those undertaken in simpler invertebrate animals such as *C*. *elegans*, can be readily performed in order to address such questions. The zebrafish genome carries four *GRN* genes [50]. Of these, *zfPGRN-A* is the orthologue of mammalian *GRN*, and is a long-form PGRN [50], encoding a protein with 10 repeats of the cysteine-rich granulin motif. *zfPGRN-B* is also a long-form PGRN, whereas *zfPGRN-1* and *zfPGRN-2* are short-form PGRNs, with one and one half granulin motifs, S1A Fig [50]. Zebrafish MNs express *zfPGRN-A* [51] and when zfPGRN-A protein levels are attenuated in developing embryos, motor function is disrupted, and the developing primary caudal (CaP) MNs show structural abnormalities, being truncated and with aberrant patterns of branching [51]. Ectopic expression of PGRN in the embryo overcomes the MN abnormalities that result from expression of mutant TDP-43 [52]. In contrast, complete deletion of zebrafish *zfPGRN-A* or *zfPGRN-B* alone or double mutants produced neither truncated CaP MN nor other neuropathological phenotypes suggesting the activation of compensatory mechanisms upon loss of *GRN* genes in the germline [53]. Similar examples of genetic compensation have been observed in other zebrafish gene knockout studies [54].

Here we take advantage of the zebrafish model of motor dysfunction to investigate the genetic interactions of PGRN with TDP-43 and FUS in a vertebrate system. We also determined whether the short form *zfPGRN-1* is able to rescue motor defects resulting from the depletion of long form *zfPGRN-A*.

## Materials and methods

### Fish husbandry

Wild type zebrafish were purchased from Aquatica Tropicals (Florida) and housed on a 14h/10h light/dark cycle at 28.5°C in a laboratory aquarium (Allentown Caging Equipment Co. Inc., Allentown, NJ). The McGill University Institutional Animal Care and Use Committee (IACUC) specifically approved this study and Zebrafish were maintained according to Protocol Number 3935. Fish were fed twice daily. In the late afternoon of the day before embryos are required (approximately 3:00 p.m.), fish were transferred to a breeding tanks with a divider to maintain male and female separately. In the morning, immediately after the light cycle begins, divider removed to facilitate spawning. After spawning has stopped, the eggs that have fallen through the breeding tanks to the outer tank were collected from the bottom of the tank. Embryos to be used for experimental studies were collected and staged by hours post fertilization (hpf) [55] and stored at recommended temperature (28.5°C) for the downstream applications.

### Microinjection

#### Microinjection of anti-sense morpholino oligonucleotides into embryos

Anti-Sense Morpholino oligonucleotides (AMO) [56] were obtained from Gene Tools, Inc. (Philomath, OR) and diluted in nuclease free water containing 0.05% phenol red or 0.05% Fast Green dye. Approximately 2 nL of AMO was injected into the yolk of 1- to 4-cell stage embryos using a PLI-100 microinjection system (Harvard Apparatus, St. Laurent, QC, Canada). Phenotype observation and images acquisition were accomplished using a Leica DC300F digital camera connected to a Leica MZFLIII stereomicroscope. Images were processed with Adobe Photoshop 7.0 software. The sequence of the AMO corresponding to the 5'UTR region of zfPGRN-A (MO2) was 5'GAGCAGGTGGATTTGTGAACAGCGG3' [51]. The sequence of the AMO corresponding to the UTR sequences of *Tardbp* (5’*GTACATCTCGGCCATCTTTCCTCAG3’*) and *Fus (**5’GGCCATAATCATTTGACGCCATGTT3’*) and standard control (5’CCTCTTACC TCAGTTACAATTTATA3’) were also used. For Morpholino injection the optimal concentration of gene- specific AMOs were determined initially and 10 ng zfPGRN-A, 10 ng of *Fus*, 1.5 ng of *Tardbp* or 10ng of standard control were used.

#### Microinjection of mRNA into 1 to 4 cell stage embryos

For zfPGRN-A mRNA over-expression and rescue experiments a full-length zfPGRN-A/pcDNA3 vector was generated [51]. Briefly, the full-length zfPGRN-A sequence was purchased from RZPD (Berlin, Germany) as clone UCDMp574E2318Q2 and subcloned into pcDNA3.1-V5/His vector (Invitrogen, Carlsbad, CA) using a forward primer that overlapped with the starter AUG and a reverse primer that read through the termination codon. The final vector construct consisted of full-length zfPGRN-A with a carboxyl-terminal tag consisting of the V5 epitope and 6×Histidine. The authenticity of the construct was verified by DNA sequencing. Other vectors used were full-length hPGRN/pcDNA3, FUS/pCS2+ or Tardbp/pCS2+. For the zfPGRN-A rescue experiments the AMO directed against the 5'UTR region of zfPGRN-A. The construct for zfPGRN-A mRNA microinjection does not contain the untranslated 5' sequence of zfPGRN-A. Hence there is no binding between mRNA and the morpholino when they are co-injected. Translation enhanced capped mRNA was synthesized with the mMessage mMachine Kit (Ambion, Huntingdon, England). For mRNA overexpression and rescue experiments 100 ng/ μl of zfPGRN-A or hPGRN or 60 ng/μl of *FUS (WT or mutant* R521H) or 25ng/ μl of *Tardbp* (WT or mutant G348C) or PGRN-1 100 ng/ μl mRNA was used. The optimal concentration of gene specific mRNAs was determined to avoid toxicity related phenotype.

### Whole-mount immunofluorescence

The znp1 immuno-labelled wild-type embryos were carried out according to previously described protocol [51]. Briefly, embryos were fixed using 4% paraformaldehyde (PFA) in phosphate buffered saline (PBS) for 2 hours at room temperature and then stored in 100% methanol at -20°C. Embryos were rehydrated with PBS and permeabilized by digesting with 10 ug/ml proteinase K for 20 minutes followed by fixation in 4% PFA / PBS for 20 minutes. After several PBS washes embryos were blocked in PBS containing 0.1% Tween-20 (PBST) with 5% calf serum and 1% DMSO. After three hours the primary antibody (anti-Znp1 ZIRC) was added at 1:200 dilution and incubated overnight at 4°C. After extensive washing in PBST, embryos were incubated with Alexa 488 anti-mouse secondary antibody. After 2 hours, embryos were washed extensively in PBST. Stained embryos were visualized with a Leica MZ FLIII stereomicroscope equipped with a GFP filter, photographed and processed with Adobe Photoshop 7.0 software.

### Touch evoked swimming behaviour

To analyse the rescue effect of PGRN in reversing MN defects due to altered expression of TDP43 or FUS at a functional level, we tested the embryo touch response and avoidance swimming behaviour at 52 hrs post injection [51, 57]. The embryos co-injected with either zfPGRN-A or hPGRN showed an improved avoidance swimming phenotype when compared to that resulting from knockdown of TDP 43 or FUS or the expression of their corresponding mutant forms. The percentages of fish showing motor deficit were determined for each condition. Their responses were recorded using a Photron (San Diego, CA) Fastcam PCI high-speed video camera.

### Analysis of Caudal Primary Motor Neurons (CaP MNs) in WT embryos

Caudal primary motor axons in whole-mounted 48 hpf wild-type embryos labelled with Znp1 monoclonal antibody. Only the trunk CaP MNs (12 pairs) were scored [58]. Embryos were counted with respect to how many of the neurons of the 12 pairs in each demonstrated a particular defect. For each treatment at least three experiments were performed and at least 50 embryos were scored per determination. Values were expressed as mean ± standard error of the mean.

### *In vivo* monitoring and imaging followed by measurement of axonal length, spinal cord length or embryo length

Embryos obtained from the HB9:GFP transgenic fish expressing GFP within the MNs following microinjection of embryos with AMOs or mRNAs or co-injection of both were visualized and photographed *in vivo*. Images were captured at 5x magnification and the hatched box was further subject to 4-5x zoom. MN axonal length from the specified areas in the spinal cord region were measured using imageJ by tracing the labelled MN axons. The spinal cord length or embryo length were also measured by tracing spinal cord or embryo using imageJ. For each treatment at least three experiments were performed and at least 50 MNs were scored per determination. Values were expressed as mean +/- standard error of the mean.

#### Statistics

Statistical significance among experimental groups was determined by one-way ANOVA followed by Student-Newman-keuls Multiple Comparisons Test (p < 0.001-***, p < 0.01-**, p < 0.05-*) using GraphPad software (GraphPad Software Inc., San Diego, CA) or one-way ANOVA followed by Holm-Sidak Multiple Comparisons Test (p < 0.05-*) using Sigma plot software (Systat Software, Inc., San Jose, CA). Error bars represent s.e.m.

### Western blotting

Zebrafish embryos were lysed using Laemmli buffer and extracts were boiled for 5 minutes. Embryo extracts were resolved using 10% acrylamide gels and transferred to nitrocellulose membrane. SDS/PAGE western blotting analyses were performed as described previously [59]using a polyclonal antibodies against PGRN-A (Sheldon Biotechnology Centre, McGill University), TDP43 (ProteinTech), FUS and a monoclonal antibody against actin (Sigma).

### RT-PCR

Zebrafish embryos were lysed in trizol and the extracted RNA was quantified. cDNA synthesis was carried out according to the manufacturer’s protocol (Fermentas) and used for gene specific PCR reactions (Invitrogen). Species-specific primer sets designed to detect zfPGRN-A mRNA employing 5'-TGATGGAACCACATGCTGTAA-3'/ 5'-CAGATGACCTCTGACCTGCTC-3’ or 5'-CTCAACTCACTCACATCCGC3'/5'-GTTTATAGAGTTAGGGCTC-3’as described in [50]. TDP-43 full and TDP-43 splice variant mRNA were 5'-CCCCATGTCTAAATGCTCTCA-3'/5'-CTTCTCACTCTTCGCCATCAC-3' or 5'-CGTCACCTTCGCAGACGATCAGGTT-3'/5'-GCCCACGATCCATCATTTGCCTACTATT-3' and 5'-CGTCACCTTCGCAGACGATCAGG TT-3'/5'-GCCTAAGCACAATAATATTCATCACCTCTTTTCCAATT-3', respectively as described in [60]. Beta-actin mRNA was used as a loading control using 5'- ATGGATGATGAAATTGCCGC-3' and 5'-TGTCATCTTTTCCCTGTTGG-3' primers and zfPGRN-A mRNA observed employing 5'-TGATGGAACCACATGCTGTAA-3'/ 5'-CAGATGACCTCTGACCTGCTC-3’ as described in [50]. Polymerase chain reaction was completed using Taq (Invitrogen) with a denaturation of 2 min at 94°C; 35 cycles at 94°C, 30 sec; 55°C or 60°C, 30 sec; 72°C, 30 sec; and a final extension of 5 min at 72°C.

## Results

### Inhibition of zfPGRN-A translation resulted in the generation of shorter axons and stalling of CaP Motor Neurons (MNs) at the horizontal myoseptum (a connective tissue structure that separates the myotome into dorsal and ventral compartments)

**Fig 1:**
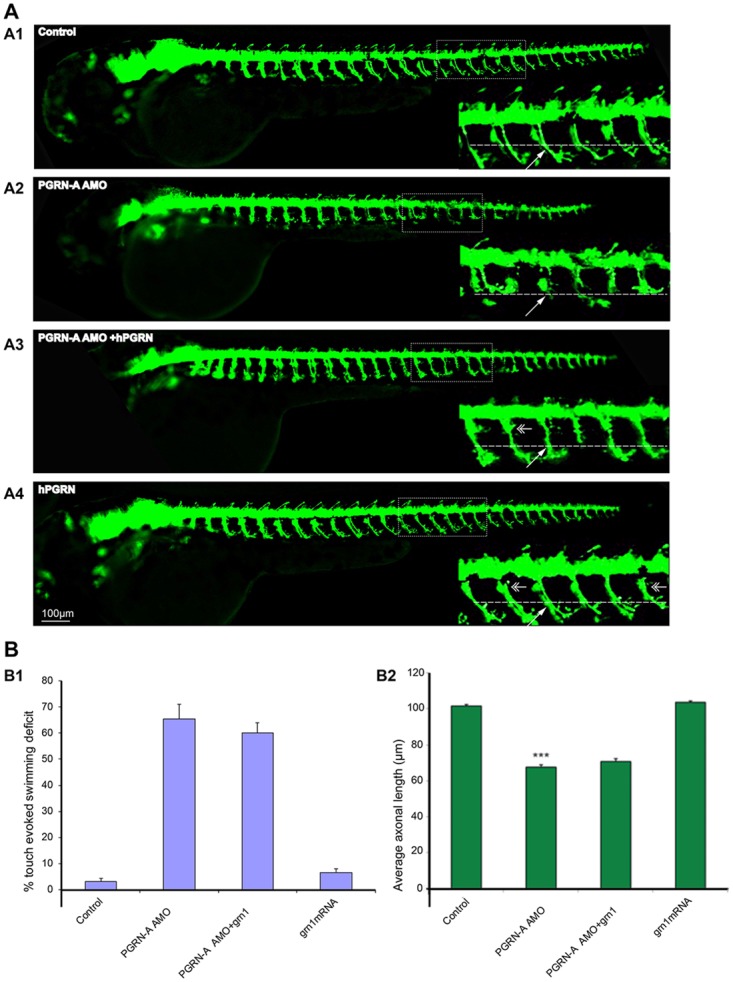
Inhibition of zfPGRN-A translation resulted in the generation of shorter axons and stalling of CaP Motor Neurons (MNs) at the horizontal myoseptum. **Fig 1A** Inhibition of zfPGRN-A translation produced truncated axons in live zebrafish. Compared to control (A1), zfPGRN-A knockdown produced shorter axons (A2) that were rescued by hPGRN mRNA (A3) while hPGRN alone did not produce truncated axons but increased branching (A4). Lateral views (anterior to the left; dorsal to the top) of embryos obtained from the Hb9:GFP transgenic fish. Images were captured at 5X magnification and the hatched box was further subject to 4-5X Zoom. Dashed lines represent the horizontal myoseptum (HM). The double- headed arrows represent increased branching and the white arrow represents axonal length.**Fig 1B** Expression of the short form zebrafish granulin zfPGRN-1 did not reverse the phenotype due to zfPGRN-A knockdown. B1. zfPGRN-1 did not rescue locomotor defects produced by the knockdown of zfPGRN-A assessed by employing touch evoked swimming test. **B2** zfPGRN-1 did not rescue motor axon defects produced by zfPGRN-A Knockdown. The ability of PGRN mRNA to rescue motor neuron defects in zebrafish is therefore limited to some but not all PGRN proteins. MN axonal length from the specified areas in the spinal cord region was measured using imageJ by tracing the labelled MN axons.

In previous work, we demonstrated that administration of the antisense morpholinos against zfPGRN-A but not control morpholinos, knock down zfPGRN-A protein expression. The protein suppression is specific for zfPGRN-A since there was no effect on levels of zf-PGRN-B, and the zfPGRN-A phenotype, namely impaired touch-sensitive motor responses, truncation of MN development and aberrant branching of MNs, is reversed by co-injection of zfPGRN-A mRNA or its human ortholog, hPGRN mRNA, but not by GFP mRNA when co-injected together with the zfPGRN-A morpholinos [51]. Here we use HB9-GFP fish that express GFP in their MNs, allowing easier tracking of MN development. We confirmed that the anti-PGRN MOs are active in the HB9-GFP line, resulting in truncated CaP MNs, and that PGRN mRNA reverses this defect as previously reported in WT zebrafish (Fig 1A). The related GRN gene zfPGRN-1, encoding a short-form GRN with only 1.5 granulin modules (S1A Fig), is unable to reverse either the touch evoked avoidance swimming behaviour at 52 hrs post injection, or the shortening of the length of the CaP MNs caused by knocking down zfPGRN-A levels (Fig 1B). zfPGRN-1 is absent or only weakly expressed in early embryos [50] and its ectopic overexpression after microinjection into the embryos was confirmed by whole mount immunofluorescence microscopy in 48 hr embryos (S1B Fig)). Not all proteins within the GRN family are therefore able to reverse the motor phenotype that arises from zfPGRN-A knockdown in the zebrafish embryo.

### Neuromuscular developmental characteristics of zfPGRN-A knockdown embryos

**Fig 2:**
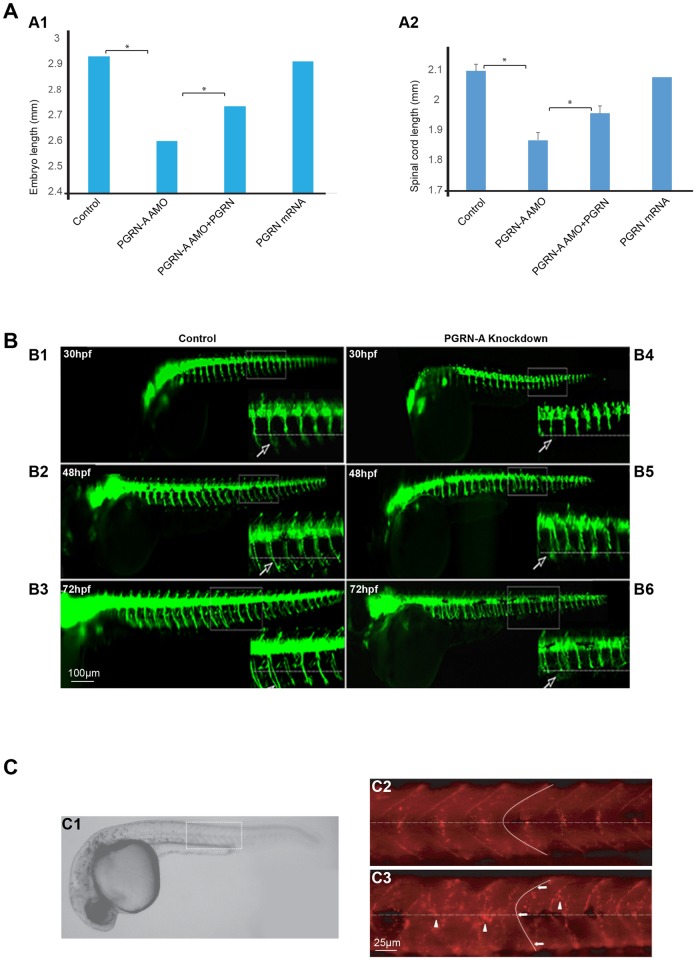
Neuromuscular developmental characteristics of zfPGRN-A knockdown embryos. **Fig 2A**. Embryos injected with zfPGRN-A AMO displayed reduced overall body length (**A1)** and a corresponding reduction in the length of the spinal cord **(A2)**. Co-injection of hPGRN mRNA with zfPGRN-A AMO (zfPGRN-A AMO+hPGRN) significantly reversed these phenotypes while hPGRN mRNA alone had no effect on overall body length or the length of the spinal cord **(Fig 2A)**. The spinal cord length or embryo length were measured by tracing spinal cord or embryo using imageJ. **Fig 2B** zfPGRN-A knockdown resulted in CaP MNss stalling at the horizontal myoseptum as shown by the failure of axonal outgrowth to extend beyond the horizontal myoseptum for up to 72hpf Controls demonstrate normal outgrowth (B1, B2, B3). In the embryos expressing a reduced level of zfPGRN-A CaP MNs are stalled at HM (B4, B5, B6). The panels show lateral views (anterior to the left; dorsal to the top) of embryos obtained from the HB9:GFP transgenic fish in which MNs express HB9 promoter-driven GFP at 30hpf (B1, B4) 48hpf (B2, B5) and 72hpf (B3, B6). Images were captured at 5X magnification and the hatched box was further subjected to 4–5 X Zoom. Dashed lines represent a horizontal myoseptum (HM). Observed phenotypes were normal MN development (Control 30hpf (B1), 48hpf (B2), 72hpf (B3); white arrows) and truncated or shorter axons (zfPGRN-A AMO 30hpf (B4), 48hpf (B5), 72hpf (B6); white arrows). **Fig 2C PGRN** PGRN deficient embryos displayed aberrant somatic boundaries as well as disorganized and mislocated AChR aggregates in the neuromuscular junctions of the somatic muscles. Panel C1 shows a lateral view of the wild type 27hpf embryo. The boxed area is representative of the areas shown in panels C2 and C3 and dashed lines indicate the horizontal myoseptum. AChR clusters were visualized by alpha-Bungarotoxin staining of control embryos at 27hpf (C2)) and in embryos injected with PGRN-A MO (C3). PGRN-A knockdown embryos show aberrant somite boundaries compared with wild type embryos as indicated by tracing of somite boundaries. PGRN-A knockdown embryos also displayed disorganized and mislocated AchR clusters AChR aggregates compared with wild type embryos. Arrows indicate somite boundaries. Arrowheads denote AChR aggregates.

We have further characterized the phenotype of zfPGRN-A knockdown animals. They display reduced overall body length and a corresponding reduction in the length of the spinal cord (Fig 2A). This indicates a general delay in development compared to control embryos. In addition, there are specific disruptions in the development of the neuromuscular unit. During development, primary MNs normally extend towards the horizontal myoseptum (a region of connective tissue separating the myotome into dorsal and ventral compartments that is present only in the embryo). At the horizontal myoseptum the axons normally contact specialized muscle pioneer cells and is a choice point for the axons where they pause before growing along separate pathways to their final targets [61]. In zfPGRN-A knockdown embryos, the extension of the CaP axons towards the horizontal myoseptum is slower than the control embryos (Fig 2B) and once they reach the horizontal myoseptum they exhibit a failure to extend ventrally beyond the horizontal myoseptum that persists for at least until 72 hrs post injection (Fig 2B). Thus, the axon truncation in zfPGRN-A knockdown fish resulted from stalling of axon outgrowth at the horizontal myoseptum, rather than delayed but otherwise normal outgrowth. The functional development of MNs requires the correct formation of neuromuscular synapses and clustering of nicotinic acetylcholine receptors (AChR) is an early event in the process [62, 63]. Alexa 594-conjugated α-bungarotoxin staining was used to detect the AChR clusters in normal and PGRN-A MO injected embryos at 27hpf. This revealed that the stereotypical pattern of AchR clustering in WT embryos [64] was severely altered as indicated by the presence of disorganized and mislocated AchR clusters in zfPGRN-A knockdown embryos, (Fig 2C), moreover, the chevron-like shape of the myotome boundaries alters, becoming less acute in the zfPGRN-A knockdown fish compared to control animals. This suggests that zfPGRN-A is required for correct formation of the neuromuscular unit.

### Knockdown and mutant expression of TDP-43 and FUS

**Fig 3:**
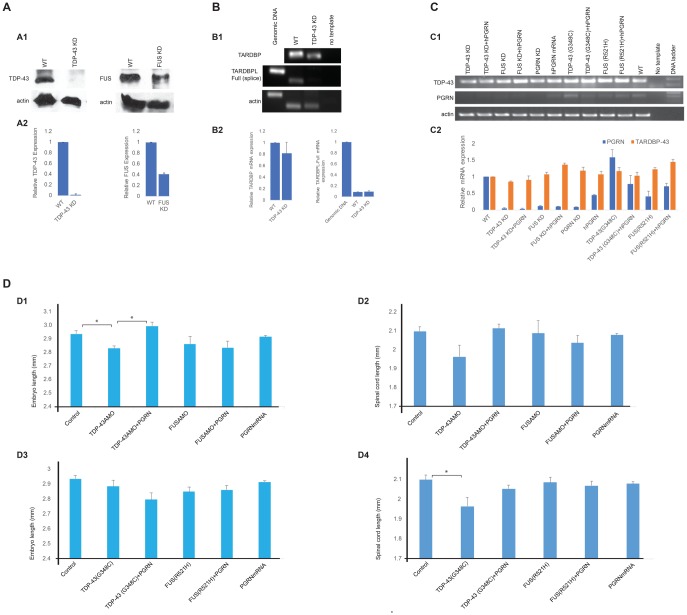
Knockdown and mutant expression of TDP-43 and FUS. **Fig 3A**. Validation of TDP-43 and FUS knockdown by Western blot analysis. The Western blot analysis of MO knockdown efficacy is a representative of three independent injection sets. Western blot analysis of protein extracts from Wild-type control embryos and embryos injected with 1.5 ng of TDP-43 AMO and 10 ng of FUS AMO (A1). Relative expression of TDP-43 and FUS with the value of wild-type set to 1.0 (A2). **Fig 3B**. RT-PCR showing knockdown of zebrafish TDP43 (Tardbp) is not associated with increased expression of mRNA for Tardbpl in the embryos (B1). Relative mRNA expression of TARDBP and TARDBPL with the value of wild-type set to 1.0 (B2). **Fig 3C**. The knockdown of TDP-43 and FUS was associated with a depletion of zfPGRN-A mRNA levels, and this was not reversed by co-expression of hPGRN (C1). Relative mRNA expression of PGRN and TARDBP with the value of wild-type set to 1.0 (C2). **Fig 3D**. Body length (D1) of the embryo and spinal cord length (D2) are reduced following the injection of TDP-43 AMO. Body length (D3) and spinal cord length (D4) of the embryo are not altered in the embryos injected with mutant FUS(R521H) while only a minor reduction in spinal cord length was observed following the injection of mutant TDP-43(G348C) (D4).

Loss of function phenotypes for zebrafish TDP-43 and FUS were generated by morpholino knockdown experiments and validated by Western blots (Fig 3A). Unlike mammalian genomes, the zebrafish genome possesses two TDP-43 related genes, *Tardbp*, which is the ortholog of the mammalian *TARDBP* gene and its paralog, *Tardbpl* (TAR DNA binding protein-like). Tardbpl lacks the glycine-rich RNA binding domain where the ALS and FTLD-U mutations occur [60]. Complete knockout of *Tardbp* can result in alternate splicing of *Tardpbl*, resulting in restoration of the glycine-rich domain allowing the alternately spliced tardbpl protein product to potentially compensate for the loss of *Tardbp* [60]. Using primers that are specific for the alternately spliced *Tardbpl* (S1 Table) we confirmed that knockdown of zebrafish TDP-43 is not associated with increased expression of mRNA for alternatively spliced Tardbpl in the embryos (Fig 3B). Tardbpl is not, therefore, a confounding factor in these experiments (Fig 3B). In contrast to *TARDBP*, there is only one *FUS* orthologue to be found in the zebrafish genome. FUS shares many structural and functional similarities with TDP 43 including the presence of a glycine-rich RNA binding domain where the mutations occur that lead to the development of ALS and FTLD-U [65]. The knockdown of TDP-43 and FUS was associated with a depletion of zfPGRN-A mRNA levels, and this was not reversed by co-expression of hPGRN (Fig 3C). We also examined the effects of over-expression of mRNA that encodes for mutants of TDP-43 and FUS that cause motor neuron disease in humans. The zfPGRN-A mRNA levels remained unchanged following expression of mutant TDP-43(G348C) or FUS(R521H) (Fig 3C). In contrast to the knockdown of zfPGRN-A, the body lengths of the embryo and spinal cord lengths are not altered in the embryos injected with FUS AMO or mutant FUS(R521H) (Fig 3D) while only a minor reduction in bodylength was observed following the knockdown of TDP-43 (TDP-43 AMO, Fig 3 panel D1) and spinal cord length following the injection of mutant TDP-43(G348C) (Fig 3 panel D4). The decreases seen with TDP-43 AMO or TDP-43(G348C) are small compared to zfPGRN-A knockdown (Fig 2A) indicating minimal development delay in these embryos.

### Neuromuscular phenotypes associated with knockdown or mutant expression of TDP-43 or FUS are compensated by the over-expression of PGRN

**Fig 4:**
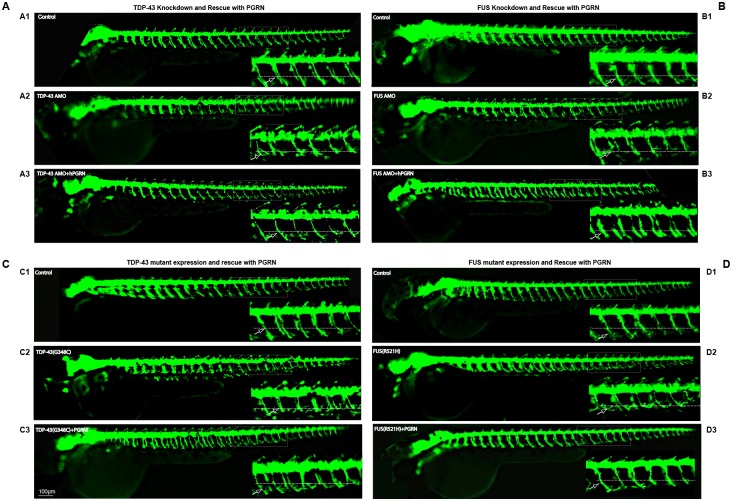
PGRN rescues motor axon defect produced by TDP-43 and FUS knockdown or expression of their mutant mRNAs. Lateral views (anterior to the left; dorsal to the top) of embryos obtained from the HB9:GFP transgenic fish. **Fig 4A**. Compared to controls (A1) embryos injected with TDP-43 AMO produced truncated axons (A2). Embryos co-injected with hPGRN mRNA (TDP-43 AMO+hPGRN) partially reversed the truncation phenotype (A3). **Fig 4B**. Compared to control (B1), embryos injected with FUS AMO produced truncated axons (B2). Embryos co-injected with hPGRN mRNA (FUS AMO +hPGRN) reversed the truncation phenotype (B3). **Fig 4C**. Compared to controls (C1), embryos injected with TDP43 (G348C) produced truncated axons (C2). Embryos co-injected with hPGRN mRNA (TDP43 (G348C)+hPGRN) reversed the truncation phenotype (C3). **Fig 4D**. Compared to controls (D1), embryos injected with FUS (R521H) produced truncated axons (D2). Embryos co-injected with hPGRN mRNA (FUS (R521H)+hPGRN) reversed the truncation phenotype (D3). Dashed line indicates the horizontal myoseptum. Arrow points to a single axon. Images were captured at 5X magnification and the hatched box was further subject to 4-5X Zoom.

We investigated whether knockdown of TDP-43 or FUS, or expression of pathogenic TDP-43(G348C) or FUS(R521H) mutants, resulted in motor neuron abnormality using HB9:GFP embryos as well as WT embryos. We have observed at the morphological level, TDP43 (Fig 4A) and FUS knockdown (Fig 4B), or expression of their mutant mRNAs (Fig 4C and 4D), resulted in shortened axons, and more frequent axon branching before the horizontal myoseptum in both HB9:GFP embryos (Fig 4) as well as WT embryos (S2 Fig). These phenotypes were also reversed by co-expression of hPGRN mRNA (Fig 4, S2 Fig).

**Fig 5:**
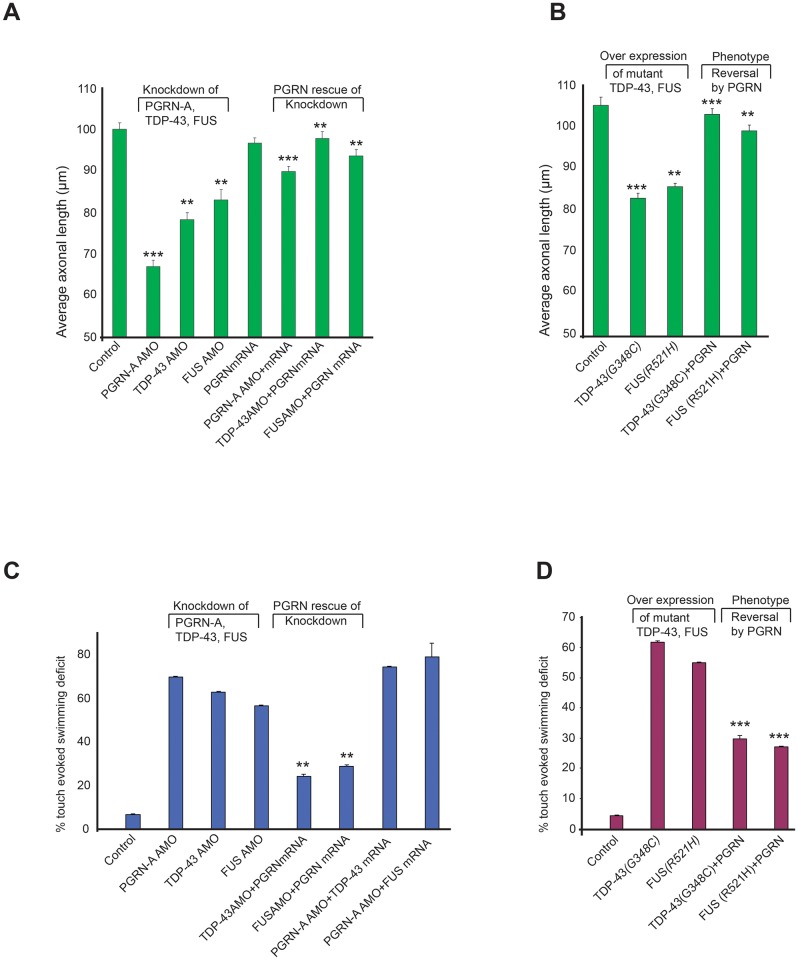
Neuromuscular phenotypes associated with knockdown or mutant expression of TDP-43 or FUS are compensated by the over-expression of PGRN. **Fig 5A** Knockdown of either TDP-43 or FUS resulted in truncated axons while co-expression of hPGRN mRNAPGRNPGRN significantly reduced axon truncation. **Fig 5B** Over-expression of either TDP-43 (*G348C*) or FUS *(R521H)* resulted in truncated axons that were rescued significantly by hPGRN mRNA. MN axonal length from the specified areas in the spinal cord region was measured using imageJ by tracing the labelled MN axons. **Fig 5C** PGRN rescues motor defects resulting from loss of function of TDP-43 or FUS. The avoidance swimming pattern was assessed by touching the larvae. Reduced expression of TDP-43 or FUS produced motor defect. PGRN co-injection reversed these defects. **Fig 5D** PGRN rescues locomotor defects due to the expression of mutants of TDP-43 and FUS. Over expression of mutant TDP-43 or FUS produced locomotor defects. PGRN co-injection significantly reversed these defects. Statistical significance was analysed by comparing knockdown of PGRN-A or TDP-43 or FUS versus the same treatments together with co-injection of PGRN mRNA respectively or by comparing expression of TDP-43(*G348C*) or FUS*(R521H)* versus the same treatments together with co-injection of PGRN mRNA respectively.

We have quantified the axonal length among different groups. Reduced expression of either TDP-43 or FUS resulted in reduction of axonal length while co-injection of either TDP-43 AMO or FUS AMO together with PGRN significantly increased axonal length (Fig 5A). In addition, over-expression of pathogenic TDP-43(G348C) or FUS(R521H) mutants resulted in shortened axons while embryos co-injected with hPGRN mRNA (TDP43 (G348C)+hPGRN) or (FUS (R521H)+hPGRN) significantly reversed truncation phenotype (Fig 5B).

We have also investigated whether morphilino knockdown of TDP-43 or FUS (Fig 5C), or expression of pathogenic TDP-43(G348C) or FUS(R521H) mutants (Fig 5D), resulted in impaired touch evoked swim responses in both HB9:GFP embryos (Fig 5C and 5D) as well as WT embryos (S2A1, S2B1, S2C1 and S2C2 Fig). At a little after 48 hrs post injection, the initial touch response where the animals flex upon contact is unaltered in TDP-43 and FUS deficient embryos. There is however, a marked defect in the subsequent strong avoidance swimming phenotype, where the embryos swim away in response to touch. This is quantified as the touch evoked swimming deficit and is a functional measurement of motor neuron defects (Fig 5C and 5D). This is consistent with the results of previous studies [66]. At 72 hrs post injection, the motility of PGRN deficient embryos is further reduced and this defect worsens progressively beyond 72 hpf (data not shown). This suggests that while there is no major sensory deficit (i.e the touch-evoked flexing is intact), there is a significant motor deficit possibly due to defective innervation of MNs in the TDP-43 knockdown embryos. The touch evoked swimming defects were significantly reversed by the co-injection of hPGRN mRNA with either the morpholinos or the mutant TDP43 or FUS mRNA (Fig 5C and 5D). The touch evoked swim deficits were reflected in structural modifications of the CaP MNs. Knockdown of either TDP-43 or FUS (Fig 5A) or expression of either TDP-43 (*G348C*) or FUS (R521H) mRNA (Fig 5B) resulted in truncated axons while co-expression of hPGRN mRNA with either the knockdown morpholinos or the mutant mRNAs resulted in significant restoration of MN average lengths.

### TDP-43 or FUS does not rescue the neuromuscular defects caused by depletion of zfPGRN-A levels

To determine whether there is complementarity between TDP-43 and FUS with GRN, we investigated whether enhanced expression of TDP-43 or FUS is able to compensate for the depletion of zfPGRN-A. Expression of WT TDP-43 or FUS was unable to reverse the touch evoked swim response or the MN structural abnormalities in the zfPGRN-A knockdown in both HB9:GFP embryos (Fig 5C) as well as WT embryos (S3A1 and S3B2 Fig).

## Discussion and conclusion

The development of CaP MNs in zebrafish embryos provides a valuable tool with which to investigate the interaction of genes that influence vertebrate MN development and function [63, 67]. In humans, haploinsufficiency of *GRN* results in a form of FTLD-U that is characterized by the accumulation of cellular inclusions of ubiquitinated-TDP-43 in affected neurons [4, 5, 68, 69]. Gene delivery of PGRN to the brain in murine models of Parkinson’s disease [2] and AD [3] prevents the onset and progression of neurodegeneration. This strongly suggests that PGRN maintains the health of the brain even when challenged by neurodegenerative stimuli that are not dependent on mutations of *GRN*. There is considerable potential for therapies based upon PGRN. In contrast, PGRN does not suppress the development of disease-like phenotypes in models of ALS that were caused by mutations of *SOD1* (*Superoxide dismutase-1*) [52, 70]. It is unclear at present the range of neuropathologies that might be responsive to the neuroprotective effect of PGRN.

Mutations in *TARDBP* represent 1–4% of familial ALS cases while mutations in *FUS* represent 4% of familial ALS cases [71]. FUS and TARDBP but not SOD1 interact in genetic models of ALS [57], raising the question as to whether and how PGRN interacts with the TDP-43/FUS axis of spinal MN degeneration. Earlier studies have indicated that PGRN overcomes MN defects in zebrafish embryos caused by ectopic expression of mutant *TARDBP* [52]. We have extended this to investigate whether defects caused by manipulation of FUS are also responsive to PGRN over expression. Furthermore, we have investigated whether phenotypes caused by loss of function of TDP-43 or FUS as well as expression of the toxic TARDBP and FUS mutants are influenced by PGRN expression. In addition, we studied whether the MN defects caused by depletion of zfPGRN-A are in turn sensitive to the over expression of WT TDP43 or FUS.

Depletion of zfPGRN-A in zebrafish embryos resulted in delayed development, as indicated by shorter body length (Fig 2A). In addition, phenotypic evidence of MN defects was observed (Figs 1A and 2B). When touched the zfPGRN-A knockdown animals flex, demonstrating that their touch-responsive sensory systems are functional, but the subsequent avoidance swimming behavior is severely curtailed. This is consistent with a defect in the motor response. During development in zebrafish, there are three primary MNs per somatic hemisegment (CaP, MiP and RoP neurons). These extend axons towards the horizontal myoseptum. On arrival, the primary MNs pause for a few hours, and then either resume axonal extension beyond the horizontal myoseptum (CaP and MiP) or grow laterally (RoP) [67]. The horizontal myoseptum is, therefore, a choice point in MN development. Branching of axons and the formation of neuromuscular junctions occur after the axons have crossed this choice point. In the zfPGRN-A knockdown embryos, the primary MN axons reach the horizontal myoseptum, but unlike in control embryos, they do not progress beyond the choice point, remaining stalled at this location for at least further 24 hours (Fig 2B). The MN truncation and branching abnormalities in zfPGRN-A knockdown embryos are therefore due to failure to progress beyond a critical choice point in MN development, rather than to a non-specific retardation in MN axonal outgrowth. What controls primary MN outgrowth beyond the horizontal myoseptum is not completely understood. The clustering of acetylcholine choline receptor (AchR) on target muscle groups is important, since mutations such as *unplugged* that interfere with AchR clustering, also disrupt the outgrowth of primary MNs beyond the horizontal myoseptum [72]. AchR cluster formation precedes MN outgrowth [73]. We found that the organization of AchR clusters was distorted in zfPGRN-A knockdown embryos compared to control animals (Fig 2C). Furthermore, the myotome somatic boundaries were misshapen relative to WT embryos. Therefore, it is likelythat zfPGRN-A knockdown is disruptive at several junctures in the development of the neuromuscular unit.

Proteolytic degradation of PGRN liberates granulin peptides, each of which is composed of a single granulin module [74]. Granulin peptides possess biological activity, although often opposite to the activity of PGRN [1]. PGRN is, for example, generally anti-inflammatory, whereas the granulin peptides are pro-inflammatory [1]. The role of granulin peptides in the brain is not well understood since most research has focused on PGRN. However, there is evidence that granulin peptides are neurotrophic [28, 75]. In contrast to other Vertebrates zebrafish have multiple *GRN* genes (S1A Fig) of which *zfGRN*-1 and *zfGRN-2*, which generate peptides with one and a half granulin modules [50]. Therefore, we enquired whether these endogenous granulin peptides of zebrafish are potentially neuroactive. The forced expression of zfPGRN-1 mRNA in early embryos was insufficient to overcome the motor defects associated with zfPGRN-A depletion (Fig 1B). We conclude that the endogenous granulin peptides of zebrafish are unable to substitute for the neuronal actions of long-form zfPGRN-A, at least in terms of CaP MN development.

The zfPGRN-A knockdown phenotype is consistently more severe than that resulting from TDP43/FUS depletion. In particular, the TDP-43 and FUS knockdown phenotypes show little difference in spinal cord and body length compared to control embryos, suggesting that no overall delay in development occurs in these embryos. Depletion of the TDP-43 and FUS, or the expression of their corresponding mutant genes, leads to the appearance of MN defects in zebrafish embryos [57]. Over expression of TDP-43 in zebrafish is not accompanied by the accumulation of TDP-43 intraneuronal cellular deposits [52]. In mice, motor defects are observed in several transgenic models of mutant or WT *TARDBP* expression even in the absence of TDP-43 aggregation, suggesting that loss of TDP-43 function, rather than toxicity of the TDP-43 aggregates, is critical in triggering TDP-43-dependent neurodegeneration [76]. The TDP-43/FUS loss of function knockdown embryos fully replicate the phenotypes following the expression of corresponding mutant genes with respect to touch evoked swim responses and axon development. Therefore, in the zebrafish partial loss of function of TDP-43 or FUS, and the pathogenicity of the mutant genes are phenotypically largely equivalent. As previously reported [52], the expression of hPGRN in zebrafish embryos reverses the motor function impairment and axon extension defects caused by the expression of mutant TDP-43. We show in the current study that the exogenous expression of hPGRN also reverses these phenotypes when caused by expression of mutant FUS, or by the depletion of TDP43 and FUS protein levels (Figs 4 and 5). zfPGRN-A is, therefore, capable of compensating for *TARDBP* and *FUS* mutation and loss of TDP-43 or FUS function. The depletion of TDP43 or FUS is accompanied by a corresponding depletion in zfPGRN-A mRNA (Fig 3C), but this is not required for the motor function phenotype since no decrease in zfPGRN-A was observed due to mutant *TARDBP* or *FUS* even though the embryos show an equivalent degree of axonopathy.

Genetic complementation studies in a *C*. *elegans* model of motor neuronopathy driven by polyQ expansion in the *huntingtin* gene identified TARDBP as upstream of *GRN* [49]. Applying the same rationale to the zebrafish results, if mutant *TARDBP*, or TDP-43 knockdown, is upstream of *GRN*, then complementation with PGRN should rescue mutant *TARDBP*, as was observed [49]. The possibility that *GRN* is upstream of *TARDBP* can be eliminated since complementation of PGRN-depleted embryos by the over-expression of TDP-43 does not rescue the effects of PGRN depletion. A similar relationship was identified in zebrafish between *FUS* and *GRN* although this relationship with FUS was not observed for *huntingtin* gene evoked neurodegeneration in *C*. *elegans* [49]. The reason for the inconsistency between the models is unknown at present. However, in terms of pathology *GRN*-dependent FTLD-U develops in the opposite sense to that deduced in gene complementation analyses, since *GRN* mutations are congenital, whereas the TDP-proteinopathy develops only at a later age. The apparent contradiction in hierarchy between gene complementation analysis and what would be expected from the natural history of *GRN*-dependent neuropathology can be resolved if *GRN* mutation in FTLD is permissive for, rather than causal of, the TDP-43 pathology of FTLD-U. In this model *GRN* mutations create a cellular environment that permits excursions of the TDP-43 system beyond their normal boundaries to manifest a toxic outcome.

S2 Table summarizes the interactions of PGRN with neuropathogenic genes and is based on data from the work presented here and by others. PGRN is unable to reduce the severity of motor phenotypes caused by the ALS gene *SOD1* or. by depleting the semaphorin co-receptor NRP1. It did, however, reduce the disease-like neuronal phenotypes caused by polyQ extension in the huntingtin protein [49]. PGRN proved notably successful in blocking the neuropathogenic RNA-binding proteins, including TDP-43 and FUS, and, as we reported previously, the spinal muscular atrophy gene, SMN-1 [51]. Whether PGRN signaling acts directly on the RNA-binding proteins, or indirectly by preventing the pathological fallout of defective RNA-processing, is uncertain and requires further research. Several groups have proposed that PGRN is neuroprotective due to its ability to restore a normalized neuroinflammatory status [25–30]. The actions of PGRN in zebrafish embryos are independent of inflammation as the appearance of acquired immune mechanisms do not occur until about four weeks of development [77, 78]. We suggest, therefore, that PGRN exerts a combined neuroprotective action, both by modulating neuroinflammation [31–41], and, as shown here, by protecting neurons directly against the harmful effects of several neurodegenerative genes. This makes PGRN a uniquely promising molecule for the development of future therapies aimed at combatting a range of neurodegenerative conditions.

## Supporting information

S1 Fig Diagrammatic representation of the mRNA encoding Zebrafish PGRN proteins and validation of PGRN-1 expression with the embryos injected with Grn1 mRNA. S1A Fig. Diagrammatic representation of the mRNA encoding Zebrafish PGRN proteins. zebrafish PGRN extend gene family with 4 *Grn* genes. *Grn A* and *Grn B* encodes PGRN protein with 9 and 10 grn motif respectively while *Grn-1* and *Grn-2* encodes PGRN protein with one and a half grn motif. S1B Fig PGRN-1 is expressed within Grn1 mRNA injected embryos not in control within 48hpf embryos.
(TIF)

S2 Fig PGRN rescues motor axon defects produced by TDP-43 or FUS knockdown in WT embryos. S2A Top panel. PGRN rescues motor axon defects produced by TDP-43 Knockdown in WT embryos. TDP-43 knockdown produced shorter axons that were rescued by co-injection of hPGRN mRNA. Lateral views (anterior to the left; dorsal to the top) of embryos labelled with znp1 mAb at 27 hpf in wild-type embryos, embryos injected with TDP-43 AMO, embryos co-injected with hPGRN mRNA (TDP-43AMO+hPGRN). Embryos co-injected with hPGRN mRNA (TDP-43AMO+hPGRN) partially reversed the truncation phenotype. Observed phenotypes were normal MN development (WT), increase in truncated and branched axons (TDP43 MO) and partial rescue of truncated MNs (TDP43 MO+PGRN). Dashed lines represent the horizontal myoseptum. S2A Fig bottom panel. PGRN rescues motor axon defects induced by FUS knockdown. FUS knockdown produced shorter axons that were rescued by hPGRN mRNA. Lateral views (anterior to the left; dorsal to the top) of embryos labelled with znp1 mAb at 27 hpf in wild type embryos, embryos injected with FUS AMO, embryos co-injected with hPGRN mRNA (FUS AMO +hPGRN). Embryos co-injected with hPGRN mRNA (FUS AMO+hPGRN) partially reversed truncation phenotype. Observed phenotypes were normal MN development (WT), increase in truncated and branched axons (FUS MO) and partial rescue of truncated MNs (FUS MO+PGRN). Dashed lines represent horizontal myoseptum. Images were captured at 20X magnification and the hatched box was further subject to 4-5X Zoom. **S2B Fig** TDP-43 knockdown and partial rescue with over-expression of PGRN mRNA in WT embryos. Average number of Truncated (B1) and Branched (B2) CaP MNs per group. S2C Fig. FUS knockdown and partial rescue with over-expression of PGRN. Average number of Branched (C1) and Truncated (C2) CaP MNs per group.
(TIF)

S3 Fig PGRN rescues motor defects due to knockdown or mutant expression of TDP-43 or FUS but not vice versa in WT embryos. **S3A Fig** PGRN rescues motor defects due to TDP-43 knockdown (A1) but not vice versa (A2) in WT embryos. Touch-evoked swimming is greatly impaired in embryos injected with TDP43 Antisense MO. The motility defect was partially but significantly rescued when the embryos were co-injected with hPGRN together with the TDP-43 antisense MO (A1). **S3B Fig** PGRN rescues motor defects due to FUS knockdown (B1) but not *vice versa* (B2) in WT embryos. Touch-evoked swimming is impaired in embryos injected with FUS antisense MO. The motility defect was partially but significantly rescued when the embryos were co-injected with hPGRN mRNA together with the FUS antisense MO (B1). **S3C Fig** PGRN rescues motor defects due to toxic gain of function induced by TDP-43(G348C) (C1) /FUS (R521H) (C2) in WT embryos. Touch-evoked swimming is greatly impaired in embryos injected with TDP43 (G348C) or FUS (R512H). The motility defect was partially but significantly rescued when the embryos were co-injected with hPGRN mRNA together with TDP43 (G348C) (C1) or FUS (R521H) (C2).
(TIF)

S1 Table NCBI database accession number for the transcript sequence used for primer design. (DOCX)

S2 Table List of genes used to knockdown or over express mutant forms and the motor phenotypes and the ability of PGRN to reverse the resultant phenotypes. (DOCX)

## References

[1] Bateman A, Bennett HP. The granulin gene family: from cancer to dementia. Bioessays. 2009;31(11):1245–54. 10.1002/bies.200900086 19795409

[2] Van Kampen JM, Baranowski D, Kay DG. Progranulin gene delivery protects dopaminergic neurons in a mouse model of Parkinson's disease. PLoS One. 2014;9(5):e97032 10.1371/journal.pone.0097032 24804730 PMC4013129

[3] Minami SS, Min SW, Krabbe G, Wang C, Zhou Y, Asgarov R, et al Progranulin protects against amyloid beta deposition and toxicity in Alzheimer's disease mouse models. Nat Med. 2014;20(10):1157–64. 10.1038/nm.3672 25261995 PMC4196723

[4] Baker M, Mackenzie IR, Pickering-Brown SM, Gass J, Rademakers R, Lindholm C, et al Mutations in progranulin cause tau-negative frontotemporal dementia linked to chromosome 17. Nature. 2006;442(7105):916–9. Epub 2006/07/25. 10.1038/nature05016 16862116

[5] Cruts M, Gijselinck I, van der Zee J, Engelborghs S, Wils H, Pirici D, et al Null mutations in progranulin cause ubiquitin-positive frontotemporal dementia linked to chromosome 17q21. Nature. 2006;442(7105):920–4. 10.1038/nature05017 16862115

[6] Gijselinck I, Van Broeckhoven C, Cruts M. Granulin mutations associated with frontotemporal lobar degeneration and related disorders: an update. Human mutation. 2008;29(12):1373–86. Epub 2008/06/11. 10.1002/humu.20785 18543312

[7] Neary D, Snowden JS, Gustafson L, Passant U, Stuss D, Black S, et al Frontotemporal lobar degeneration: a consensus on clinical diagnostic criteria. Neurology. 1998;51(6):1546–54. 9855500 10.1212/wnl.51.6.1546

[8] Seelaar H, Rohrer JD, Pijnenburg YA, Fox NC, van Swieten JC. Clinical, genetic and pathological heterogeneity of frontotemporal dementia: a review. J Neurol Neurosurg Psychiatry. 2011;82(5):476–86. 10.1136/jnnp.2010.212225 20971753

[9] Rohrer JD, Guerreiro R, Vandrovcova J, Uphill J, Reiman D, Beck J, et al The heritability and genetics of frontotemporal lobar degeneration. Neurology. 2009;73(18):1451–6. 10.1212/WNL.0b013e3181bf997a 19884572 PMC2779007

[10] Rohrer JD, Nicholas JM, Cash DM, van Swieten J, Dopper E, Jiskoot L, et al Presymptomatic cognitive and neuroanatomical changes in genetic frontotemporal dementia in the Genetic Frontotemporal dementia Initiative (GENFI) study: a cross-sectional analysis. Lancet Neurol. 2015;14(3):253–62. 10.1016/S1474-4422(14)70324-2 25662776 PMC6742501

[11] DeJesus-Hernandez M, Mackenzie IR, Boeve BF, Boxer AL, Baker M, Rutherford NJ, et al Expanded GGGGCC hexanucleotide repeat in noncoding region of C9ORF72 causes chromosome 9p-linked FTD and ALS. Neuron. 2011;72(2):245–56. 10.1016/j.neuron.2011.09.011 21944778 PMC3202986

[12] Renton AE, Majounie E, Waite A, Simon-Sanchez J, Rollinson S, Gibbs JR, et al A hexanucleotide repeat expansion in C9ORF72 is the cause of chromosome 9p21-linked ALS-FTD. Neuron. 2011;72(2):257–68. 10.1016/j.neuron.2011.09.010 21944779 PMC3200438

[13] Gass J, Cannon A, Mackenzie IR, Boeve B, Baker M, Adamson J, et al Mutations in progranulin are a major cause of ubiquitin-positive frontotemporal lobar degeneration. Hum Mol Genet. 2006;15(20):2988–3001. 10.1093/hmg/ddl241 16950801

[14] Pickering-Brown SM, Richardson AM, Snowden JS, McDonagh AM, Burns A, Braude W, et al Inherited frontotemporal dementia in nine British families associated with intronic mutations in the tau gene. Brain: a journal of neurology. 2002;125(Pt 4):732–51. Epub 2002/03/26. 11912108 10.1093/brain/awf069

[15] Neumann M, Sampathu DM, Kwong LK, Truax AC, Micsenyi MC, Chou TT, et al Ubiquitinated TDP-43 in frontotemporal lobar degeneration and amyotrophic lateral sclerosis. Science. 2006;314(5796):130–3. 10.1126/science.1134108 17023659

[16] Rademakers R, Neumann M, Mackenzie IR. Advances in understanding the molecular basis of frontotemporal dementia. Nat Rev Neurol. 2012;8(8):423–34. 10.1038/nrneurol.2012.117 22732773 PMC3629543

[17] Mackenzie IR, Bigio EH, Ince PG, Geser F, Neumann M, Cairns NJ, et al Pathological TDP-43 distinguishes sporadic amyotrophic lateral sclerosis from amyotrophic lateral sclerosis with SOD1 mutations. Ann Neurol. 2007;61(5):427–34. 10.1002/ana.21147 17469116

[18] Brouwers N, Bettens K, Gijselinck I, Engelborghs S, Pickut BA, Van Miegroet H, et al Contribution of TARDBP to Alzheimer's disease genetic etiology. J Alzheimers Dis. 2010;21(2):423–30. 10.3233/JAD-2010-100198 20555136

[19] Mackenzie IR, Neumann M, Bigio EH, Cairns NJ, Alafuzoff I, Kril J, et al Nomenclature for neuropathologic subtypes of frontotemporal lobar degeneration: consensus recommendations. Acta Neuropathol. 2009;117(1):15–8. 10.1007/s00401-008-0460-5 19015862 PMC2710877

[20] Deng HX, Zhai H, Bigio EH, Yan J, Fecto F, Ajroud K, et al FUS-immunoreactive inclusions are a common feature in sporadic and non-SOD1 familial amyotrophic lateral sclerosis. Ann Neurol. 2010;67(6):739–48. 10.1002/ana.22051 20517935 PMC4376270

[21] Sreedharan J, Blair IP, Tripathi VB, Hu X, Vance C, Rogelj B, et al TDP-43 mutations in familial and sporadic amyotrophic lateral sclerosis. Science. 2008;319(5870):1668–72. 10.1126/science.1154584 18309045 PMC7116650

[22] Kabashi E, Valdmanis PN, Dion P, Spiegelman D, McConkey BJ, Vande Velde C, et al TARDBP mutations in individuals with sporadic and familial amyotrophic lateral sclerosis. Nat Genet. 2008;40(5):572–4. 10.1038/ng.132 18372902

[23] Huey ED, Ferrari R, Moreno JH, Jensen C, Morris CM, Potocnik F, et al FUS and TDP43 genetic variability in FTD and CBS. Neurobiol Aging. 33(5):1016 e9–17. Epub 2011/09/29. 10.1016/j.neurobiolaging.2011.08.004 PMC4489700 21943958

[24] Lefebvre S, Burglen L, Reboullet S, Clermont O, Burlet P, Viollet L, et al Identification and characterization of a spinal muscular atrophy-determining gene. Cell. 1995;80(1):155–65. 7813012 10.1016/0092-8674(95)90460-3

[25] Gao X, Joselin AP, Wang L, Kar A, Ray P, Bateman A, et al Progranulin promotes neurite outgrowth and neuronal differentiation by regulating GSK-3beta. Protein Cell. 2010;1(6):552–62. 10.1007/s13238-010-0067-1 21204008 PMC4875315

[26] Guo A, Tapia L, Bamji SX, Cynader MS, Jia W. Progranulin deficiency leads to enhanced cell vulnerability and TDP-43 translocation in primary neuronal cultures. Brain Res. 2010;1366:1–8. 10.1016/j.brainres.2010.09.099 20888804

[27] Ryan CL, Baranowski DC, Chitramuthu BP, Malik S, Li Z, Cao M, et al Progranulin is expressed within motor neurons and promotes neuronal cell survival. BMC Neurosci. 2009;10:130 10.1186/1471-2202-10-130 19860916 PMC2779192

[28] Van Damme P, Van Hoecke A, Lambrechts D, Vanacker P, Bogaert E, van Swieten J, et al Progranulin functions as a neurotrophic factor to regulate neurite outgrowth and enhance neuronal survival. J Cell Biol. 2008;181(1):37–41. 10.1083/jcb.200712039 18378771 PMC2287280

[29] Kleinberger G, Wils H, Ponsaerts P, Joris G, Timmermans JP, Van Broeckhoven C, et al Increased caspase activation and decreased TDP-43 solubility in progranulin knockout cortical cultures. J Neurochem. 2010;115(3):735–47. 10.1111/j.1471-4159.2010.06961.x 20731760

[30] Xu J, Xilouri M, Bruban J, Shioi J, Shao Z, Papazoglou I, et al Extracellular progranulin protects cortical neurons from toxic insults by activating survival signaling. Neurobiol Aging. 2011;32(12):2326 e5–16. 10.1016/j.neurobiolaging.2011.06.017 PMC3375317 21820214

[31] Moisse K, Volkening K, Leystra-Lantz C, Welch I, Hill T, Strong MJ. Divergent patterns of cytosolic TDP-43 and neuronal progranulin expression following axotomy: implications for TDP-43 in the physiological response to neuronal injury. Brain Res. 2009;1249:202–11. 10.1016/j.brainres.2008.10.021 19046946

[32] Philips T, De Muynck L, Thu HN, Weynants B, Vanacker P, Dhondt J, et al Microglial upregulation of progranulin as a marker of motor neuron degeneration. J Neuropathol Exp Neurol. 2010;69(12):1191–200. 10.1097/NEN.0b013e3181fc9aea 21107132

[33] Tanaka Y, Matsuwaki T, Yamanouchi K, Nishihara M. Exacerbated inflammatory responses related to activated microglia after traumatic brain injury in progranulin-deficient mice. Neuroscience. 2013;231:49–60. 10.1016/j.neuroscience.2012.11.032 23201826

[34] Zhu S, Tai C, Petkau TL, Zhang S, Liao C, Dong Z, et al Progranulin promotes activation of microglia/macrophage after pilocarpine-induced status epilepticus. Brain Res. 2013;1530:54–65. 10.1016/j.brainres.2013.07.023 23887054

[35] Yin F, Banerjee R, Thomas B, Zhou P, Qian L, Jia T, et al Exaggerated inflammation, impaired host defense, and neuropathology in progranulin-deficient mice. J Exp Med. 2010;207(1):117–28. 10.1084/jem.20091568 20026663 PMC2812536

[36] Kao AW, Eisenhut RJ, Martens LH, Nakamura A, Huang A, Bagley JA, et al A neurodegenerative disease mutation that accelerates the clearance of apoptotic cells. Proc Natl Acad Sci U S A. 2011;108(11):4441–6. 10.1073/pnas.1100650108 21368173 PMC3060230

[37] Martens LH, Zhang J, Barmada SJ, Zhou P, Kamiya S, Sun B, et al Progranulin deficiency promotes neuroinflammation and neuron loss following toxin-induced injury. J Clin Invest. 2012;122(11):3955–9. 10.1172/JCI63113 23041626 PMC3484443

[38] Yin F, Dumont M, Banerjee R, Ma Y, Li H, Lin MT, et al Behavioral deficits and progressive neuropathology in progranulin-deficient mice: a mouse model of frontotemporal dementia. FASEB J. 2010;24(12):4639–47. 10.1096/fj.10-161471 20667979 PMC2992364

[39] Petkau TL, Neal SJ, Milnerwood A, Mew A, Hill AM, Orban P, et al Synaptic dysfunction in progranulin-deficient mice. Neurobiol Dis. 2012;45(2):711–22. 10.1016/j.nbd.2011.10.016 22062772

[40] Filiano AJ, Martens LH, Young AH, Warmus BA, Zhou P, Diaz-Ramirez G, et al Dissociation of frontotemporal dementia-related deficits and neuroinflammation in progranulin haploinsufficient mice. J Neurosci. 2013;33(12):5352–61. 10.1523/JNEUROSCI.6103-11.2013 23516300 PMC3740510

[41] Ghoshal N, Dearborn JT, Wozniak DF, Cairns NJ. Core features of frontotemporal dementia recapitulated in progranulin knockout mice. Neurobiol Dis. 2012;45(1):395–408. 10.1016/j.nbd.2011.08.029 21933710 PMC3225509

[42] Wils H, Kleinberger G, Pereson S, Janssens J, Capell A, Van Dam D, et al Cellular ageing, increased mortality and FTLD-TDP-associated neuropathology in progranulin knockout mice. J Pathol. 2012;228(1):67–76. 10.1002/path.4043 22733568

[43] Tanaka Y, Chambers JK, Matsuwaki T, Yamanouchi K, Nishihara M. Possible involvement of lysosomal dysfunction in pathological changes of the brain in aged progranulin-deficient mice. Acta Neuropathol Commun. 2014;2:78 10.1186/s40478-014-0078-x 25022663 PMC4149276

[44] Zhou X, Sun L, Bastos de Oliveira F, Qi X, Brown WJ, Smolka MB, et al Prosaposin facilitates sortilin-independent lysosomal trafficking of progranulin. J Cell Biol. 2015;210(6):991–1002. 10.1083/jcb.201502029 26370502 PMC4576858

[45] Belzil VV, Gendron TF, Petrucelli L. RNA-mediated toxicity in neurodegenerative disease. Mol Cell Neurosci. 2013;56:406–19. 10.1016/j.mcn.2012.12.006 23280309 PMC3791208

[46] Ling JP, Pletnikova O, Troncoso JC, Wong PC. TDP-43 repression of nonconserved cryptic exons is compromised in ALS-FTD. Science. 2015;349(6248):650–5. 10.1126/science.aab0983 26250685 PMC4825810

[47] Colombrita C, Zennaro E, Fallini C, Weber M, Sommacal A, Buratti E, et al TDP-43 is recruited to stress granules in conditions of oxidative insult. J Neurochem. 2009;111(4):1051–61. 10.1111/j.1471-4159.2009.06383.x 19765185

[48] Zheng M, Shi Y, Fan D. Nuclear TAR DNA-binding protein 43: A new target for amyotrophic lateral sclerosis treatment. Neural Regen Res. 2013;8(35):3284–95. 10.3969/j.issn.1673-5374.2013.35.003 25206650 PMC4145946

[49] Tauffenberger A, Chitramuthu BP, Bateman A, Bennett HP, Parker JA. Reduction of polyglutamine toxicity by TDP-43, FUS and progranulin in Huntington's disease models. Hum Mol Genet. 2013;22(4):782–94. 10.1093/hmg/dds485 23172908

[50] Cadieux B, Chitramuthu BP, Baranowski D, Bennett HP. The zebrafish progranulin gene family and antisense transcripts. BMC Genomics. 2005;6:156 10.1186/1471-2164-6-156 16277664 PMC1310530

[51] Chitramuthu BP, Baranowski DC, Kay DG, Bateman A, Bennett HP. Progranulin modulates zebrafish motoneuron development in vivo and rescues truncation defects associated with knockdown of Survival motor neuron 1. Mol Neurodegener. 2010;5:41 10.1186/1750-1326-5-41 20946666 PMC2974670

[52] Laird AS, Van Hoecke A, De Muynck L, Timmers M, Van den Bosch L, Van Damme P, et al Progranulin is neurotrophic in vivo and protects against a mutant TDP-43 induced axonopathy. PLoS One. 2010;5(10):e13368 10.1371/journal.pone.0013368 20967127 PMC2954192

[53] Solchenberger B, Russell C, Kremmer E, Haass C, Schmid B. Granulin knock out zebrafish lack frontotemporal lobar degeneration and neuronal ceroid lipofuscinosis pathology. PLoS One. 2015;10(3):e0118956 10.1371/journal.pone.0118956 25785851 PMC4365039

[54] Rossi A, Kontarakis Z, Gerri C, Nolte H, Holper S, Kruger M, et al Genetic compensation induced by deleterious mutations but not gene knockdowns. Nature. 2015;524(7564):230–3. 10.1038/nature14580 26168398

[55] Kimmel CB, Ballard WW, Kimmel SR, Ullmann B, Schilling TF. Stages of embryonic development of the zebrafish. Dev Dyn. 1995;203(3):253–310. 10.1002/aja.1002030302 8589427

[56] Nasevicius A, Ekker SC. Effective targeted gene 'knockdown' in zebrafish. Nat Genet. 2000;26(2):216–20. 10.1038/79951 11017081

[57] Kabashi E, Bercier V, Lissouba A, Liao M, Brustein E, Rouleau GA, et al FUS and TARDBP but not SOD1 interact in genetic models of amyotrophic lateral sclerosis. PLoS Genet. 2011;7(8):e1002214 10.1371/journal.pgen.1002214 21829392 PMC3150442

[58] Feldner J B T, Goishi K, Schweitzer J, Lee P, Schachner M, Klagsbrun M, Becker CG. Neuropilin-1a is involved in trunk motor axon outgrowth in embryonic zebrafish. Developmental Dynamics. 2005;234(3):535–49. 10.1002/dvdy.20520 16110501

[59] Chitramuthu BP, Baranowski DC, Kay DG, Bateman A, Bennett HP. Progranulin modulates zebrafish motoneuron development in vivo and rescues truncation defects associated with knockdown of Survival motor neuron 1. Mol Neurodegener. 5:41 Epub 2010/10/16. 10.1186/1750-1326-5-41 20946666 PMC2974670

[60] Hewamadduma CA, Grierson AJ, Ma TP, Pan L, Moens CB, Ingham PW, et al Tardbpl splicing rescues motor neuron and axonal development in a mutant tardbp zebrafish. Hum Mol Genet. 2013;22(12):2376–86. 10.1093/hmg/ddt082 23427147 PMC3658164

[61] Bassett DI, Currie PD. The zebrafish as a model for muscular dystrophy and congenital myopathy. Hum Mol Genet. 2003;12 Spec No 2:R265–70. 14504264 10.1093/hmg/ddg279

[62] Huh KH, Fuhrer C. Clustering of nicotinic acetylcholine receptors: from the neuromuscular junction to interneuronal synapses. Mol Neurobiol. 2002;25(1):79–112. 10.1385/MN:25:1:079 11890459

[63] Flanagan-Steet H, Fox MA, Meyer D, Sanes JR. Neuromuscular synapses can form in vivo by incorporation of initially aneural postsynaptic specializations. Development. 2005;132(20):4471–81. 10.1242/dev.02044 16162647

[64] Drapeau P, Buss RR, Ali DW, Legendre P, Rotundo RL. Limits to the development of fast neuromuscular transmission in zebrafish. J Neurophysiol. 2001;86(6):2951–6. 11731551 10.1152/jn.2001.86.6.2951

[65] Mackenzie IR, Rademakers R, Neumann M. TDP-43 and FUS in amyotrophic lateral sclerosis and frontotemporal dementia. Lancet Neurol. 2010;9(10):995–1007. 10.1016/S1474-4422(10)70195-2 20864052

[66] Kabashi E, Lin L, Tradewell ML, Dion PA, Bercier V, Bourgouin P, et al Gain and loss of function of ALS-related mutations of TARDBP (TDP-43) cause motor deficits in vivo. Human molecular genetics. 2010;19(4):671–83. Epub 2009/12/05. 10.1093/hmg/ddp534 19959528

[67] Feldner J, Reimer MM, Schweitzer J, Wendik B, Meyer D, Becker T, et al PlexinA3 restricts spinal exit points and branching of trunk motor nerves in embryonic zebrafish. J Neurosci. 2007;27(18):4978–83. 10.1523/JNEUROSCI.1132-07.2007 17475806 PMC6672091

[68] Mackenzie IR, Baker M, Pickering-Brown S, Hsiung GY, Lindholm C, Dwosh E, et al The neuropathology of frontotemporal lobar degeneration caused by mutations in the progranulin gene. Brain. 2006;129(Pt 11):3081–90. 10.1093/brain/awl271 17071926

[69] Josephs KA, Ahmed Z, Katsuse O, Parisi JF, Boeve BF, Knopman DS, et al Neuropathologic features of frontotemporal lobar degeneration with ubiquitin-positive inclusions with progranulin gene (PGRN) mutations. J Neuropathol Exp Neurol. 2007;66(2):142–51. 10.1097/nen.0b013e31803020cf 17278999

[70] Herdewyn S, De Muynck L, Van Den Bosch L, Robberecht W, Van Damme P. Progranulin does not affect motor neuron degeneration in mutant SOD1 mice and rats. Neurobiol Aging. 2013;34(10):2302–3. 10.1016/j.neurobiolaging.2013.03.027 23608112

[71] Kinsley L, Siddique T. Amyotrophic Lateral Sclerosis Overview In: Pagon RA, Adam MP, Ardinger HH, Wallace SE, Amemiya A, Bean LJH, et al, editors. GeneReviews(R). Seattle (WA) 1993.

[72] Zhang J, Lefebvre JL, Zhao S, Granato M. Zebrafish unplugged reveals a role for muscle-specific kinase homologs in axonal pathway choice. Nat Neurosci. 2004;7(12):1303–9. 10.1038/nn1350 15543140

[73] Panzer JA, Song Y, Balice-Gordon RJ. In vivo imaging of preferential motor axon outgrowth to and synaptogenesis at prepatterned acetylcholine receptor clusters in embryonic zebrafish skeletal muscle. J Neurosci. 2006;26(3):934–47. 10.1523/JNEUROSCI.3656-05.2006 16421313 PMC6675385

[74] Bateman A, Belcourt D, Bennett H, Lazure C, Solomon S. Granulins, a novel class of peptide from leukocytes. Biochem Biophys Res Commun. 1990;173(3):1161–8. 2268320 10.1016/s0006-291x(05)80908-8

[75] De Muynck L, Herdewyn S, Beel S, Scheveneels W, Van Den Bosch L, Robberecht W, et al The neurotrophic properties of progranulin depend on the granulin E domain but do not require sortilin binding. Neurobiol Aging. 2013;34(11):2541–7. 10.1016/j.neurobiolaging.2013.04.022 23706646

[76] Liu YC, Chiang PM, Tsai KJ. Disease animal models of TDP-43 proteinopathy and their pre-clinical applications. Int J Mol Sci. 2013;14(10):20079–111. 10.3390/ijms141020079 24113586 PMC3821604

[77] Trede NS, Langenau DM, Traver D, Look AT, Zon LI. The use of zebrafish to understand immunity. Immunity. 2004;20(4):367–79. 15084267 10.1016/s1074-7613(04)00084-6

[78] Renshaw SA, Loynes CA, Trushell DM, Elworthy S, Ingham PW, Whyte MK. A transgenic zebrafish model of neutrophilic inflammation. Blood. 2006;108(13):3976–8. 10.1182/blood-2006-05-024075 16926288

