# Supplementary figures and images for: Neurotrophic effects of progranulin in vivo in reversing motor neuron defects caused by over or under expression of TDP-43 or FUS
Source: PLoS One. 2017 Mar 30;12(3):e0174784. doi: 10.1371/journal.pone.0174784 (PMC5373598; doi:10.1371/journal.pone.0174784)

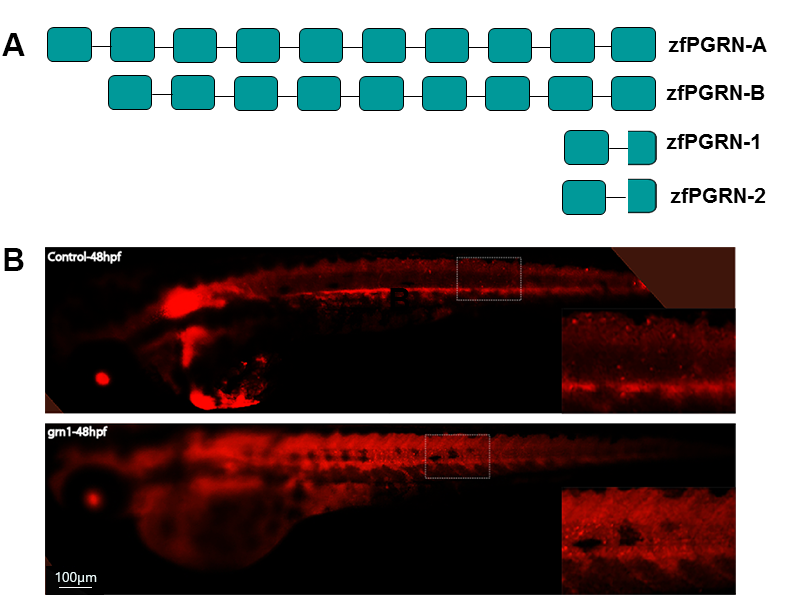

Supplement: S1 Fig — S1A Fig. Diagrammatic representation of the mRNA encoding Zebrafish PGRN proteins. zebrafish PGRN extend gene family with 4 Grn genes. Grn A and Grn B encodes PGRN protein with 9 and 10 grn motif respectively while Grn-1 and Grn-2 encodes PGRN protein with one and a half grn motif. S1B Fig PGRN-1 is expressed within Grn1 mRNA injected embryos not in control within 48hpf embryos. (TIF) [file pone.0174784.s001.tif]

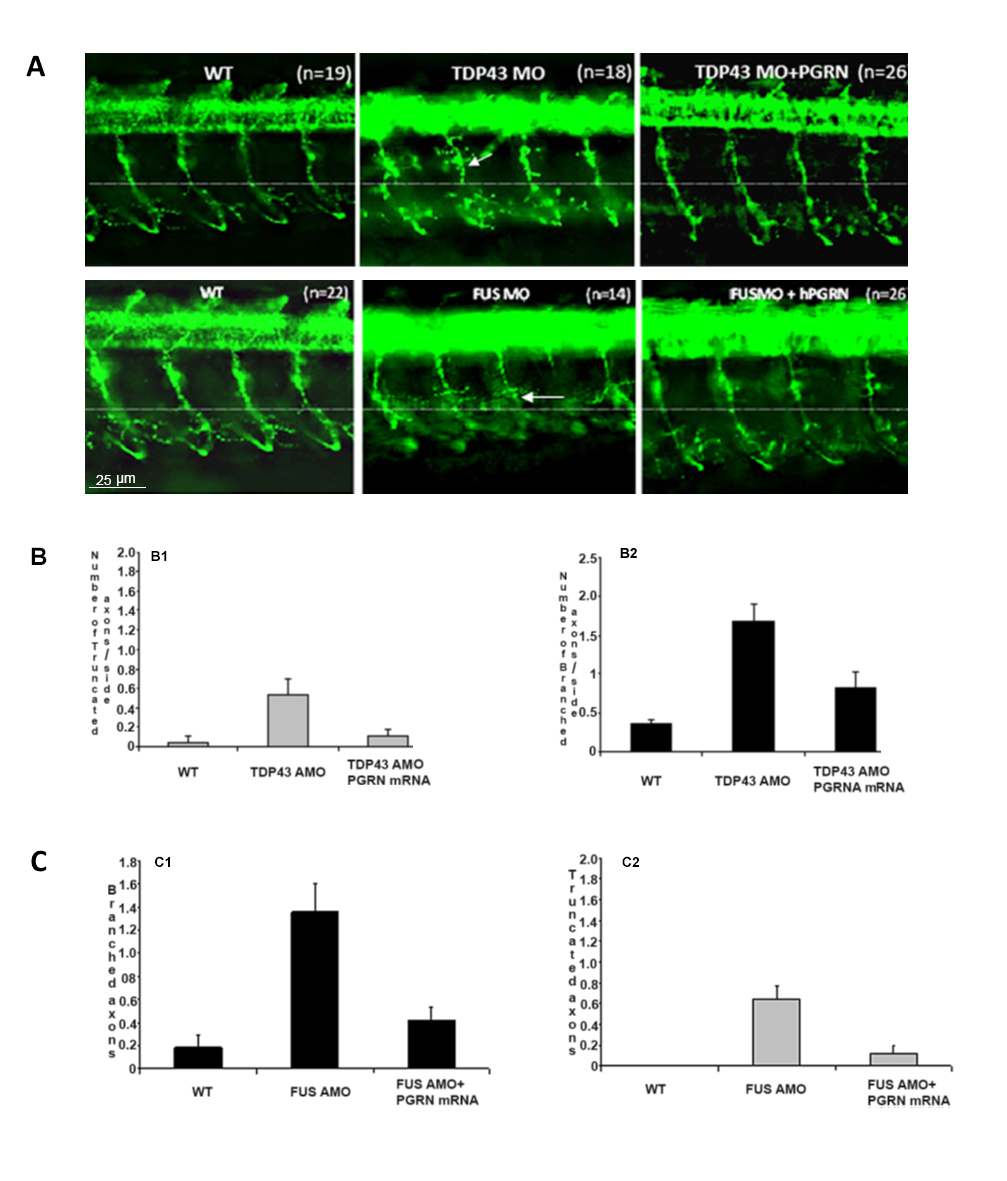

Supplement: S2 Fig — S2A Top panel. PGRN rescues motor axon defects produced by TDP-43 Knockdown in WT embryos. TDP-43 knockdown produced shorter axons that were rescued by co-injection of hPGRN mRNA. Lateral views (anterior to the left; dorsal to the top) of embryos labelled with znp1 mAb at 27 hpf in wild-type embryos, embryos injected with TDP-43 AMO, embryos co-injected with hPGRN mRNA (TDP-43AMO+hPGRN). Embryos co-injected with hPGRN mRNA (TDP-43AMO+hPGRN) partially reversed the truncation phenotype. Observed phenotypes were normal MN development (WT), increase in truncated and branched axons (TDP43 MO) and partial rescue of truncated MNs (TDP43 MO+PGRN). Dashed lines represent the horizontal myoseptum. S2A Fig bottom panel. PGRN rescues motor axon defects induced by FUS knockdown. FUS knockdown produced shorter axons that were rescued by hPGRN mRNA. Lateral views (anterior to the left; dorsal to the top) of embryos labelled with znp1 mAb at 27 hpf in wild type embryos, embryos injected with FUS AMO, embryos co-injected with hPGRN mRNA (FUS AMO +hPGRN). Embryos co-injected with hPGRN mRNA (FUS AMO+hPGRN) partially reversed truncation phenotype. Observed phenotypes were normal MN development (WT), increase in truncated and branched axons (FUS MO) and partial rescue of truncated MNs (FUS MO+PGRN). Dashed lines represent horizontal myoseptum. Images were captured at 20X magnification and the hatched box was further subject to 4-5X Zoom. S2B Fig TDP-43 knockdown and partial rescue with over-expression of PGRN mRNA in WT embryos. Average number of Truncated (B1) and Branched (B2) CaP MNs per group. S2C Fig. FUS knockdown and partial rescue with over-expression of PGRN. Average number of Branched (C1) and Truncated (C2) CaP MNs per group. (TIF) [file pone.0174784.s002.tif]

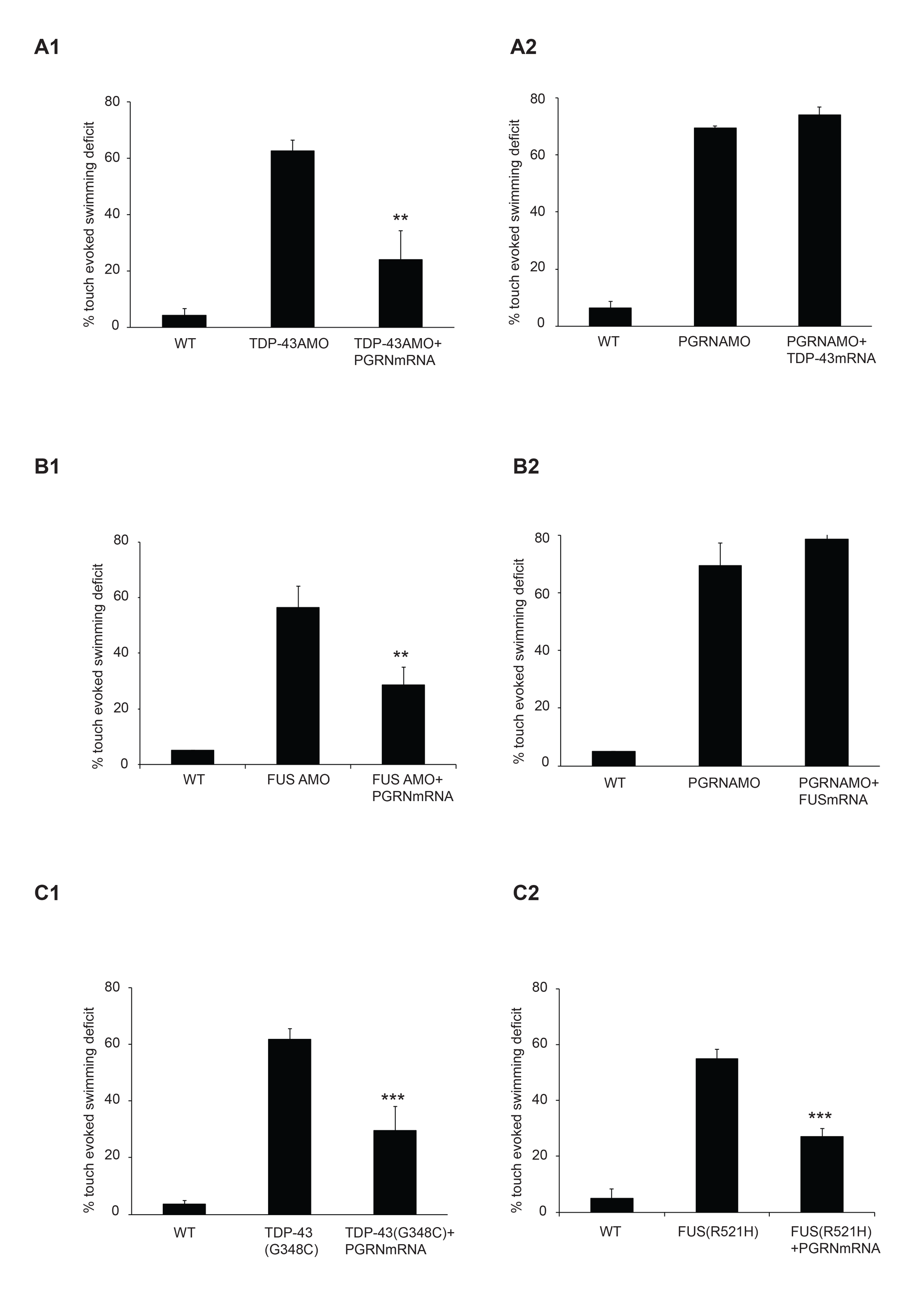

Supplement: S3 Fig — S3A Fig PGRN rescues motor defects due to TDP-43 knockdown (A1) but not vice versa (A2) in WT embryos. Touch-evoked swimming is greatly impaired in embryos injected with TDP43 Antisense MO. The motility defect was partially but significantly rescued when the embryos were co-injected with hPGRN together with the TDP-43 antisense MO (A1). S3B Fig PGRN rescues motor defects due to FUS knockdown (B1) but not vice versa (B2) in WT embryos. Touch-evoked swimming is impaired in embryos injected with FUS antisense MO. The motility defect was partially but significantly rescued when the embryos were co-injected with hPGRN mRNA together with the FUS antisense MO (B1). S3C Fig PGRN rescues motor defects due to toxic gain of function induced by TDP-43(G348C) (C1) /FUS (R521H) (C2) in WT embryos. Touch-evoked swimming is greatly impaired in embryos injected with TDP43 (G348C) or FUS (R512H). The motility defect was partially but significantly rescued when the embryos were co-injected with hPGRN mRNA together with TDP43 (G348C) (C1) or FUS (R521H) (C2). (TIF) [file pone.0174784.s003.tif]
