# Supplementary material for: Neurotrophic effects of progranulin in vivo in reversing motor neuron defects caused by over or under expression of TDP-43 or FUS
Source: PLoS One. 2017 Mar 30;12(3):e0174784. doi: 10.1371/journal.pone.0174784 (PMC5373598; doi:10.1371/journal.pone.0174784)
Supplement: S1 Table — (DOCX) [file pone.0174784.s004.docx]

**S1_Table. NCBI database accession number for the transcript sequence used for primer design.**

|  | Gene name | Accession number |
| --- | --- | --- |
| 1. | actin | NM_181601 |
| 2. | PGRN-A | NM_001001949 |
| 3. | Tardbp | NM_201476 |
| 4. | Tardbpl variant | XM_005162240 |
