# Supplementary material for: Neurotrophic effects of progranulin in vivo in reversing motor neuron defects caused by over or under expression of TDP-43 or FUS
Source: PLoS One. 2017 Mar 30;12(3):e0174784. doi: 10.1371/journal.pone.0174784 (PMC5373598; doi:10.1371/journal.pone.0174784)
Supplement: S2 Table — (DOCX) [file pone.0174784.s005.docx]

**S2 Table. List of genes modulated either by knockdown or mutant expression and the ability of PGRN to rescue the associated phenotype in zebrafish.**

| **GENE Knockdown (KD)/ mutant (mut)** | **Rescued by PGRN** | **Rescued by**  **zfPGRN-1** | **Reference** |
| --- | --- | --- | --- |
| *Grn-A* KD | + |  | [51, 52], this article |
| *Grn-A* KD |  | - | This article |
| *TARDBP -43* KD | + |  | This article |
| *FUS* KD | + |  | This article |
| *SMN-1* KD | + |  | [51] |
| TDP-43 (A315T)/(G348C) | + |  | [52], this article |
| FUS (R521H) | + |  | This article |
| *SOD1*(A4V) | - |  | [52] |
| *NRP-1*KD | - |  | [51] |
